# Processing time affects sequential memory performance beginning at the level of visual encoding

**DOI:** 10.1371/journal.pone.0265719

**Published:** 2022-03-23

**Authors:** Ryoken Takase, Jared Boasen, Shinya Kuriki, Akira Toyomura, Koichi Yokosawa

**Affiliations:** 1 Graduate School of Health Sciences, Hokkaido University, Sapporo, Hokkaido, Japan; 2 Faculty of Health Sciences, Hokkaido University, Sapporo, Hokkaido, Japan; 3 Tech3Lab, HEC Montréal, Montréal, Quebec, Canada; 4 Graduate School of Health Sciences, Gunma University, Maebashi, Gunma, Japan; Universiti Tunku Abdul Rahman, MALAYSIA

## Abstract

Electrophysiological studies have demonstrated that theta-band activity is useful for investigating neural mechanisms of memory. However, mechanisms specifically driving memory performance remain poorly understood. In sequential memory, performance can be artificially attenuated by shortening the inter-stimulus interval (ISI) between memory item presentations. Therefore, we sought to clarify the mechanisms of sequential memory performance by analyzing theta-band (4–8 Hz) activity recorded via magnetoencephalogram in 33 participants during performance of a sequential memory task where memory items were presented at either slow or fast rates in accordance with longer or shorter ISIs, respectively. Particularly in the slow task, theta activity clearly modulated in accordance with the presentation of memory items. Common cortical target regions in the occipital and frontal cortex were identified in both tasks and related to visual encoding and memory maintenance, respectively. Compared to the slow task, occipital-theta activity was significantly lower in the fast task from the midterm until the ending of encoding, in correspondence with significantly lower recall for memory items in this same period. Meanwhile, despite a loss of clarity in responsiveness to individual memory items in the fast task, frontal-theta activity was not different between tasks and exhibited particularly strong responses in both tasks during the holding period prior to recall. Our results indicate that shorter processing time erodes sequential memory performance beginning at the level of visual encoding.

## 1. Introduction

Memory is a fundamental cognitive function that is essential for daily life. Short-term memory is one kind of memory function which can be considered to occur in three stages: encoding, maintenance, and recall [1]. First, information to be memorized is encoded via sensory brain regions (e.g., visual cortex). Next, the information is transmitted to other brain regions where the information is maintained. This process of encoding and maintenance occurs concurrently until the memorized information is recalled or is no longer needed.

Numerous tasks have been used to study short-term memory, including those involving sequential memory [2–8]. The associated burden from concurrent encoding and maintenance processing is reflected in sequential memory recall performance. Memory items presented in the beginning and ending parts of the sequence are recalled with high accuracy, while items presented in the middle of the sequence are difficult to recall. This results in a U-shaped accuracy curve in accordance with an item’s position in the sequence, a phenomenon known as the serial position effect [9, 10]. In addition to serial position, sequential memory performance can also be attenuated by other factors, such as increasing the number of memory items or the complexity of the memory item characteristics, and decreasing the time interval between memory item presentations. Manipulation of these kinds of performance-dependent factors has been reported in neurophysiological studies regarding memory function [11, 12]. Among these studies, the modalities of electroencephalography (EEG) and magnetoencephalography (MEG) [8, 13–16], which permit investigation of various kinds of spontaneous brain rhythms [2, 3, 17–19] are prominently used.

There are numerous spontaneous brain rhythms which can be measured with MEG and EEG. Of these rhythms, theta-rhythm (4–8 Hz) has been shown to have particular relevance to behavioral memory performance [4, 13, 20, 21]. Looking across neurophysiological studies regarding working memory, theta-rhythm appears to play different roles in memory processing depending on the brain regions from which it originates. For example, some studies demonstrated that increased occipital-theta activity after stimulus presentation reflected visual processing of task-related stimuli [5, 22, 23]. Alternatively, Chou et al. (2015) suggested that increased occipital-theta activity reflects neural efficiency to reduce memory load [3]. Meanwhile, there have been numerous papers reporting a relationship between working memory and frontal-theta activity [4, 6–8, 13–16, 24–26]. Itthipuripat et al. (2013) indicated that increased frontal-theta activity reflected successful short-term memory maintenance [7]. Onton at al. (2005) and Maurer et al. (2014) reported that frontal-theta increases during memory maintenance [4, 8]. Other studies have shown that frontal-theta increases as a function of memory load [8, 13–16]. Together, this evidence indicates that theta activity in general reflects multiple functional aspects of memory processing across various cortical regions, especially the occipital and frontal brain regions, and should be sensitive to manipulation of performance-based factors.

Of the factors which have been reported to affect sequential memory performance, the present study focused on the inter-stimulus interval (ISI) between memory item presentation [27]. We designed the experiment to reproduce a commonly experienced real-life phenomenon where a stream of information that is presented too quickly (i.e. with an excessively short ISI) becomes difficult to memorize. Our aim was to explore and identify the stage in the memory process that ISI shortening affects neural processing. We recorded MEG during a visual sequential memory task with two different ISIs between memory item presentations. An initial investigation of this work confirmed the importance of theta oscillatory activity (5–7 Hz) via time-frequency analysis [28]. In the present report, we expand our investigation by focusing in detail specifically on the relationship between theta activity and memory performance.

Sequential memory tasks in which encoding and maintenance occur concurrently can be considered reflective of working memory in general. A consensus is still lacking as to which brain region–the prefrontal cortex or sensory areas–is responsible for encoding and storing working memory information [29, 30]. Based on our results, we discuss brain activities of the frontal region and the occipital–visual sensory–region. Our analyses of theta activity clarify which region is more affected by ISI shortening in correlation to memory performance. Thereby, this work contributes insight into the neural mechanisms of memory performance decline.

## 2. Methods

### 2.1 Participants

Thirty-three healthy students (22.8 ± 1.8 years old; all right-handed; 17 men and 16 women) were recruited from our institution to participate in this study. The experimental procedures were approved by the Ethics Committee of the School of Medicine and Faculty of Health Sciences, Hokkaido University, and written informed consent was obtained from each participant prior to the experiment.

### 2.2 MEG device and experimental setup

MEG signals were recorded with a 101-channel magnetometer system (customized; Elekta-Neuromag Oy, Helsinki, Finland) installed in Hokkaido University, Sapporo, Japan. The visual stimuli were projected by a liquid-crystal projector located outside a magnetically shielded room onto a rear-projection screen located in the magnetically shielded room.

### 2.3 Experimental tasks

**Fig 1:**
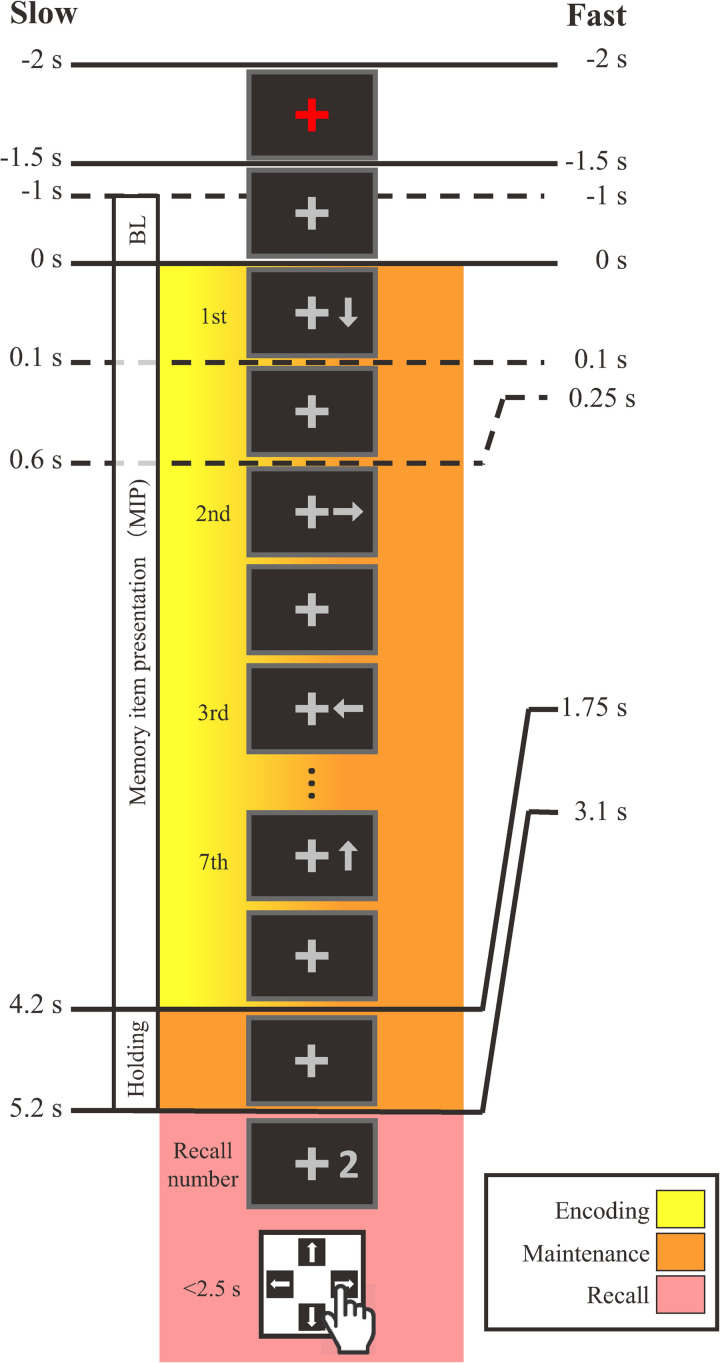
Schematic diagram of a trial of the slow (left) and fast (right) sequential memory tasks. In both tasks, seven arrows were presented sequentially as memory items. The inter-stimulus intervals between the presentation onset of each arrow in the slow and fast conditions were 0.6 s and 0.25 s, respectively. Upon presentation of a recall number cue, each participant answered the arrow direction corresponding to the recall number by pressing a button on a keypad. The color gradient indicates that both encoding and maintenance processing were concurrent during the memory item presentation period. BL: Baseline.

The present study employed two versions of a sequential memory task that we have used in prior research [31, 32]. The two versions of the task were identical in design except that the rate of memory item presentation was either slow or fast (Fig 1, left or right, respectively) due to longer or shorter ISIs, respectively. As we were interested in changes in spontaneous activity, all visual stimuli were dark gray and appeared on a black background in order to suppress transient brain responses. Additionally, all stimuli were presented within the central visual field at visual angles of 2.2°–2.8° from a cross-shaped fixation target that was continuously presented at the center of the projection screen.

Each trial in both tasks commenced with the color of the fixation target changing from gray to red for 0.5 s as a start cue. The fixation target then returned to gray for one second. Then, seven arrows, randomly directed either right, left, up, or down, appeared sequentially on the right side of the fixation target. The presentation duration of each arrow in both tasks was 0.1 s. The ISI between the offset and onset of arrow presentations in the fast task was 0.15 s and was based on preliminary experiments [28] where it was adjusted such that its time length was less than half that of the slow task while still producing accuracies above chance level (25%). The ISI between the offset and onset of arrow presentations in the slow task was 0.5 s, and was based on our prior studies using this sequential memory task [31, 32].

After the 7th arrow disappeared, the fixation target was displayed alone for 1.5 s in both tasks. Then a numerical (1–7) recall cue was presented, with the number corresponding to the serial position of a presented arrow. Recall numbers were presented in a pseudo-random manner such that all numbers appeared equally in total. The recall number remained presented until the participant answered the direction of the arrow corresponding to the recall number by pressing one of four directional response buttons with her/his right index finger as soon as possible. For example, in Fig 1, the recall number is “2”, so the participant should answer “right”, the direction of the second arrow. Participant responses for each trial were recorded automatically. With four response options, the chance accuracy level was 25%. The moment the response button was pressed, the recall number disappeared, and response feedback of either “○” (correct) or “×” (incorrect) was presented for one second to the right of the fixation cross. An inter-trial interval was randomized between three to four seconds to avoid synchronization to periodic ambient noise.

### 2.4 Experimental procedures

After greeting the participant, the experimenter provided an explanation of the task in detail and asked him/her to complete the Japanese edition of the Edinburgh Handedness Inventory [33]. Next, the experimenter digitized the location of the head position indicator coils, fiducials, and hundreds of head surface points according to standard MEG operating procedure [34]. The experimenter then guided the participant inside the magnetically shielded room and positioned her/him upright in a hydraulic chair in the MEG device. The participant then practiced several trials of the task until she/he could achieve several consecutively correct trials. The experimenter finally instructed the participant to keep her/his head, trunk, or other extremities stationary, and only move her/his right index finger to press the response button after the recall cue was presented. A camcorder monitored the inside of the magnetically shielded room constantly for safety.

The experiment was divided into two halves. The slow task was presented in one half and lasted 25 minutes. The fast task was presented in the other half and lasted 20 minutes. The order of task presentation was randomized between participants to control for order effects. The slow and fast tasks were furthermore divided into two sessions each. Thus, there were four sessions in each experiment, with a one-minute break between each session. Each session comprised 56 consecutive trials of the corresponding task. Thus, there were 224 total trials per experiment for each participant: 112 for the slow task, and 112 for fast task. In the 112 trials of each task, each recall number (1–7) appeared 16 times. Including breaks and initial practice sessions, the total time each participant spent installed in the MEG was around 50 minutes. The mean response accuracy for each participant was calculated for each task according to each recall number for use in statistical analyses of behavior.

### 2.5 MEG recording and data processing

#### 2.5.1 Artifact cleaning and epoching

MEGs were recorded at a 600 Hz sampling rate, with a passband set at 0.1–100 Hz. The MEG recordings of each participant were processed using Brainstorm [35]. The following data processing was nearly identical to that described by Boasen et al. [36]. Briefly, physiological artifacts and periodic noise were isolated and removed using independent component analysis (ICA) based on the Infomax method, one of the default ICA methods included in Brainstorm. A FIR band-pass filter was then applied at 1–30 Hz. The cleaned and band-pass filtered signals were then epoched relative to trial onset (denoted as 0 s in Fig 1) at -2–7 s for the slow task and -2–5 s for the fast task. Note that MEG data recorded after recall number presentation were not analyzed due to the inherent complications of associated motor activity from the participant [31].

#### 2.5.2 Whole-head sensor-level theta-rhythm

Theta-band (5–7 Hz, the default setting in Brainstorm software) activity envelopes were computed by Hilbert transform. The envelopes were averaged across epochs in each participant for each task type. Finally, the theta activity envelopes were averaged across all sensor signals in each participant and standardized as amplitude deviations from baseline (-1–0 s) based on the following equation,

$$x_{std}(t)=\frac{x(t)-\mu }{\mu },$$ (1)

where *x* denotes the amplitude of the non-standardized theta activity envelope at time sample *t*, and *μ* denotes the mean amplitude of the theta activity envelope over the baseline period. The time courses of the amplitude of the resulting standardized theta activity envelopes *x*_*std*_ (*t*) were averaged across participants and used in gross examination of sensor-level theta activity in each task.

#### 2.5.3 Cortical theta activity

In order to estimate cortical-level theta activity, head points and fiducial positions of each participant were co-registered on a template brain. An overlapping-sphere forward model was computed. Current dipoles were estimated for each epoch in the frequency band 1–30 Hz using minimum-norm estimation (MNE) without orientation constraints on 15002 vertices of the cortical surface. The cortical activity of each current dipole in each epoch was then decomposed to the theta band via Hilbert transform. The resulting cortical theta activities were then averaged across all epochs in each task. Finally, mean cortical theta activity for each task was standardized using Eq (1).

Two time-windows were set based on the memory task: the memory item presentation (MIP) period (slow task: 0–4.2 s; fast task: 0–1.75 s), and the holding period which occurred 1 s before the onset of the recall number (slow task: 4.2–5.2 s; fast task: 2.1–3.1 s). Furthermore, in order to investigate cortical theta activity corresponding to serial position effects, the MIP period was further divided into three sub-periods: a) the beginning sub-period corresponding to the 1st and 2nd arrows (slow task: 0–1.2 s; fast task 0–0.5 s), b) the midterm sub-period corresponding to the 3rd to 5th arrows (slow task: 1.2–3.0 s; fast task: 0.5–1.25 s), and c) the ending sub-period corresponding the 6th and 7th arrows (slow task: 3.0–4.2 s, fast task: 1.25–1.75 s). The mean standardized cortical theta activity for each task in each participant was averaged over each of the MIP sub-periods, and the holding period, to be used in cortical target area identification.

#### 2.5.4 Cortical target area identification and processing

Cortical target areas were explored by statistically analyzing standardized cortical theta activity at the group level for each period and sub-period in each task separately using one sample t-tests against zero, and illustrating the results on the template brain with a false discovery rate (FDR) of 5% (q < 0.05). Target regions for further analysis were based on the full MIP period and holding period, and were determined by highlighting the vertices for which standardized cortical theta activity was statistically positive above baseline and overlapped for both the slow and fast tasks. Returning to mean standardized cortical theta activity time-courses for each task, the time-courses were averaged across the vertices in the selected target areas, and then averaged over time for the MIP period, the MIP sub-periods, and the holding period for use in statistical analyses.

#### 2.5.5 Timing of response peaks

To gain further functional insight on the theta response dynamics in the cortical target areas, we analyzed the mean theta activity time-courses in each area for each task in each participant separately, and identified the timing of the peak theta response following the presentation of the arrows. At the individual participant level in the slow task, individual peaks were discernable in both cortical target areas in response to the presentation of all seven arrows. Using a time window of 0–0.6 s (i.e. arrow presentation plus the ISI) where 0 s is the onset of arrow presentation, the time point of peak theta response amplitude was identified for each memory item. At the individual participant level in the fast task, only peak responses to the first arrow were discernable in the cortical target areas. Therefore, the time point of peak amplitude of the theta response to the presentation of just the first arrow was identified. These extracted time points were used in subsequent statistical analyses.

### 2.6 Statistical analyses

First, memory performance was compared between men and women via two-way repeated measures analysis of variance (RM ANOVA) of overall task accuracy based on the within-subject factor of task (fast vs. slow), and the between-subject factor of sex (men vs. women). As there were no effects of sex on overall task performance (see next section), men and women were aggregated for all further analyses. Mean accuracies for each recall number in each task were analyzed via two-way RM ANOVA (task (2) × recall number (7)) in order to examine the behavioral effect of task and recall number on accuracy. The effect of task on standardized cortical theta activity during the MIP sub-periods and holding period was similarly analyzed via two-way RM ANOVA (task (2) × period/sub-period (4)) for each cortical target region separately. In the case of a significant interaction, simple main effects testing was performed to further compare between tasks. Finally, the timing of the observable peak theta responses corresponding to the presentation of arrows in the standardized theta activity time-courses in the cortical target areas were statistically compared for each task separately via paired t-test. All statistical analyses were performed using SPSS (IBM, Armonk, NY, USA). Significance thresholds were set at p < 0.05.

## 3. Results

MEG data of four participants were excluded due to excessive ambient noise or malfunction of the MEG device. Thus, the data of 29 participants were analyzed (22.9 ± 1.9 years old; 16 men and 13 women).

### 3.1 Memory performance

With respect to overall task accuracy, two-way RM ANOVA revealed a significant main effect for task (*F*_(1, 26)_ = 55.917, *p* < 0.001, *η*^2^ = 0.097). However, there was no main effect of sex on overall accuracy (*F*_(1, 26)_ = 0.810, *p* = 0.376, *η*^2^ = 0.012), nor was there a significant interaction between task and sex on overall accuracy (*F*_(1, 26)_ = 0.408, *p* = 0.529, *η*^2^ = 0.001). Therefore, men and women were aggregated for all further analyses.

**Fig 2:**
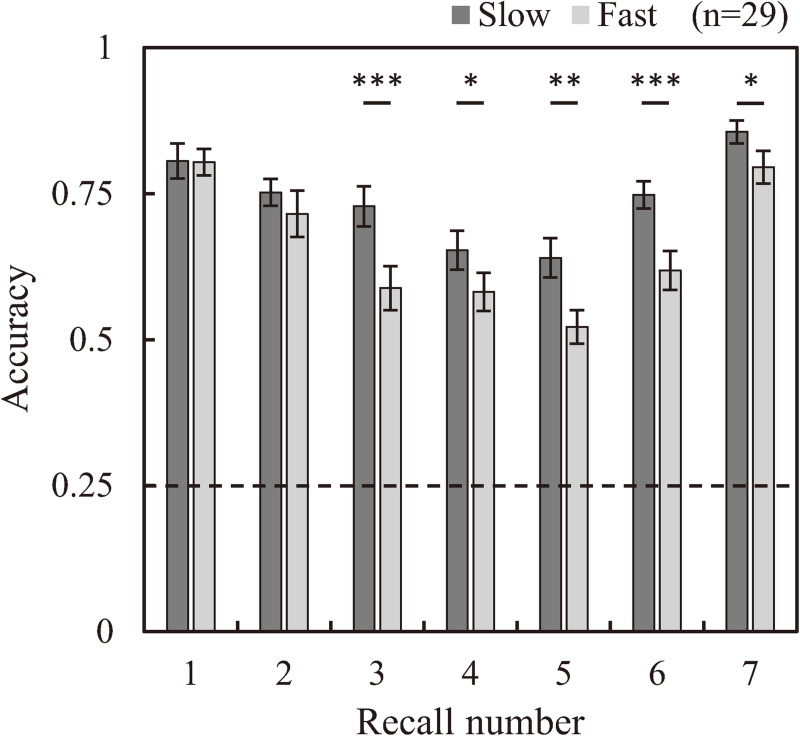
Comparison of recall accuracy between the slow and fast tasks according to each recall number. Significant differences in accuracies between tasks were observed for the direction of arrows (memory items) presented from the third to the last position in the sequence. The dashed line denotes the chance accuracy level in both tasks of 0.25. (n = 33, Error bar: SE, *: p < 0.05, **: p < 0.01, ***: p < 0.001).

Fig 2 shows mean accuracies for the slow and fast tasks for each recall number. Accuracy was above 25% chance level in all cases. Both tasks exhibited typical U-shaped accuracy curves, meaning that the direction of the arrows which were presented in beginning and ending sub-periods of the sequence were memorized better than the direction of the arrows presented in the midterm sub-period. Two-way RM ANOVA of accuracy revealed a significant interaction between task and recall number (*F*_(6, 168)_ = 2.592, *p* = 0.039, *η*^2^ = 0.085), and significant main effects for both task and recall number (*F*_(1, 28)_ = 49.072, *p* < 0.001, *η*^2^ = 0.637; *F*_(6, 168_ = 16.682, *p* < 0.001, *η*^2^ = 0.373; respectively). The main effect of task revealed that the overall mean accuracy of the fast task was significantly lower than that of the slow task (mean ± SD: 0.661 ± 0.016 vs. 0.740 ± 0.019, respectively). Simple main effects testing revealed significantly lower accuracy for the fast task compared to the slow task for arrows presented in midterm and ending sub-periods (3rd–7th recall number) (1st: *p* = 0.946; 2nd: *p* = 0.237; 3rd: *p* < 0.001; 4th: *p* = 0.045; 5th: *p* = 0.003; 6th: *p* < 0.001; 7th: *p* = 0.025).

### 3.2 Theta activity

#### 3.2.1 Sensor-level theta activity time-courses

**Fig 3:**
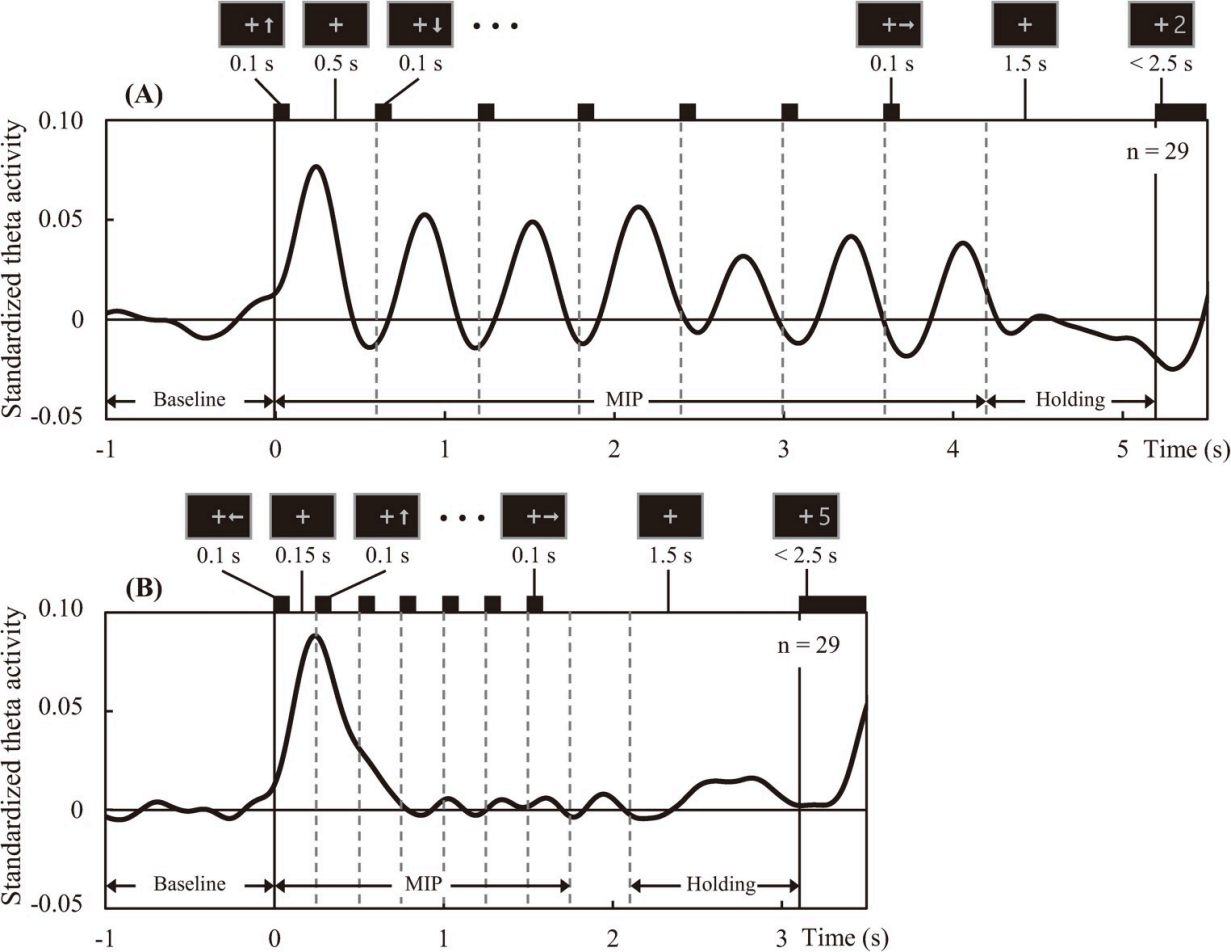
Mean time courses of standardized theta activity across all sensors; in the slow task (A) and fast task (B). Theta activity amplitude was standardized based on mean activity over the baseline period. Vertical lines indicate the onset times of the arrows (memory items) and recall numbers. Durations of presentation and some examples are shown above each time course.

Fig 3(A) and 3(B) shows the standardized theta activity time-courses of the slow and fast tasks averaged over all participants and all sensors. In the slow task, there are clear peaks of theta synchronization following the presentation of each memory item during the MIP period. Conversely in the fast task, marked theta synchronization occurs only after presentation of the first memory item.

#### 3.2.2 Whole-brain source-level differences in theta activity

**Fig 4:**
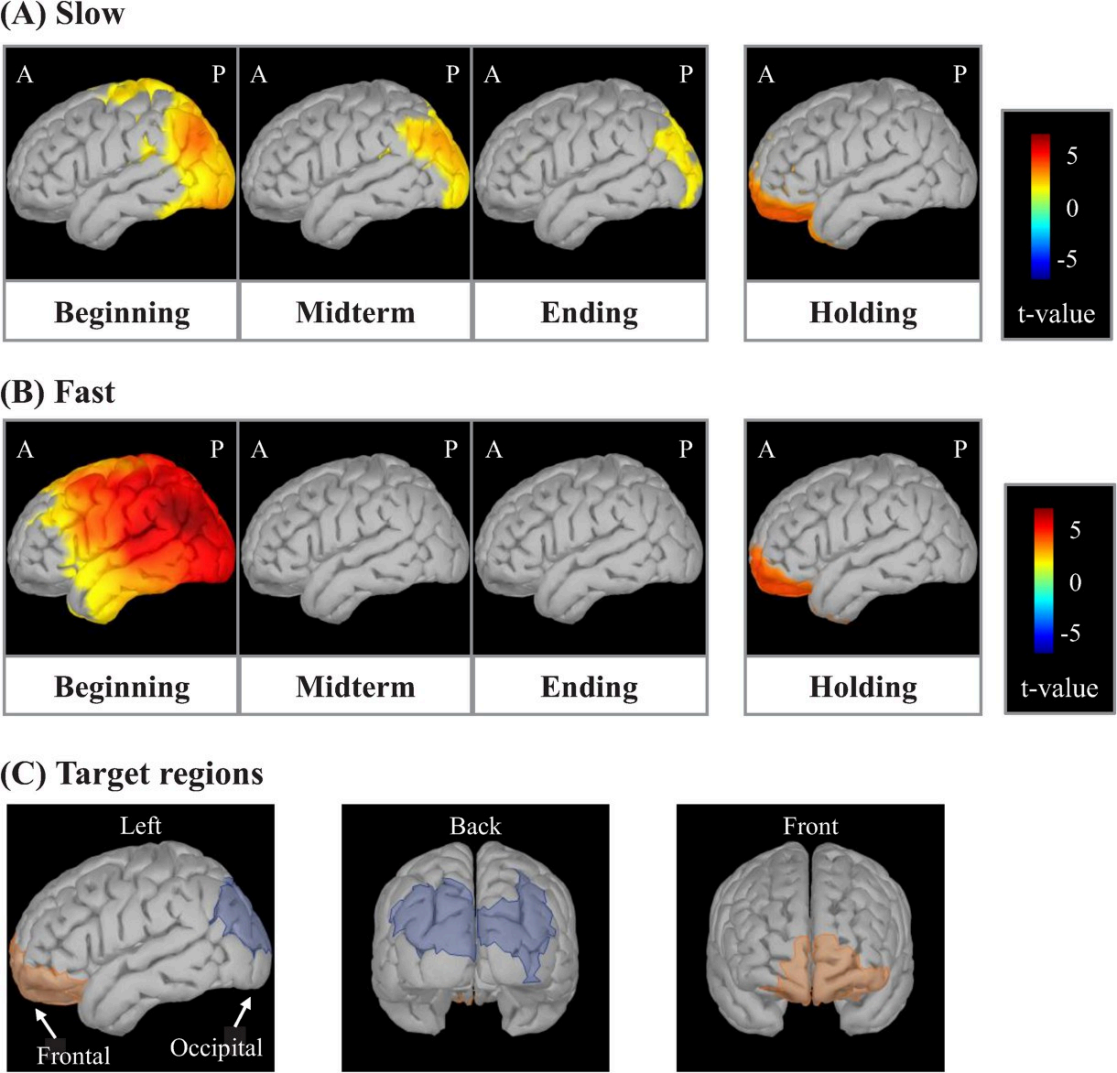
Cortical t-maps showing regions of significant standardized cortical theta activity according to task period/sub-period. Occipital-theta activity is observed throughout all sub-periods in the memory item presentation (MIP) period in the slow task (A), while prominent only in the beginning sub-period in the fast task (B). Frontal-theta is significantly active during the holding period in both tasks. The occipital and frontal regions were selected as target regions due to their significantly positive standardized cortical theta activities in both tasks during the MIP and holding periods, respectively (C).

Fig 4(A) and 4(B) shows cortical t-maps for the slow and fast task during the beginning, midterm and ending MIP sub-periods, and the holding period. The cortical t-maps for the slow task revealed that significant t-values were positive, albeit progressively decreasing in area, across all three MIP sub-periods in the occipital region. Meanwhile, the cortical t-maps for the fast task also revealed significantly positive t-values in the occipital region, but only during the beginning sub-period, with no positive area observed in the midterm and ending sub-periods. The cortical t-maps also revealed significantly positive t-values in the frontal region for both tasks during the holding period. As discussed in the methods section, these occipital and frontal cortical areas were targeted for subsequent statistical analyses of standardized cortical theta activity.

#### 3.2.3 Theta activity time-courses in targeted brain regions

**Fig 5:**
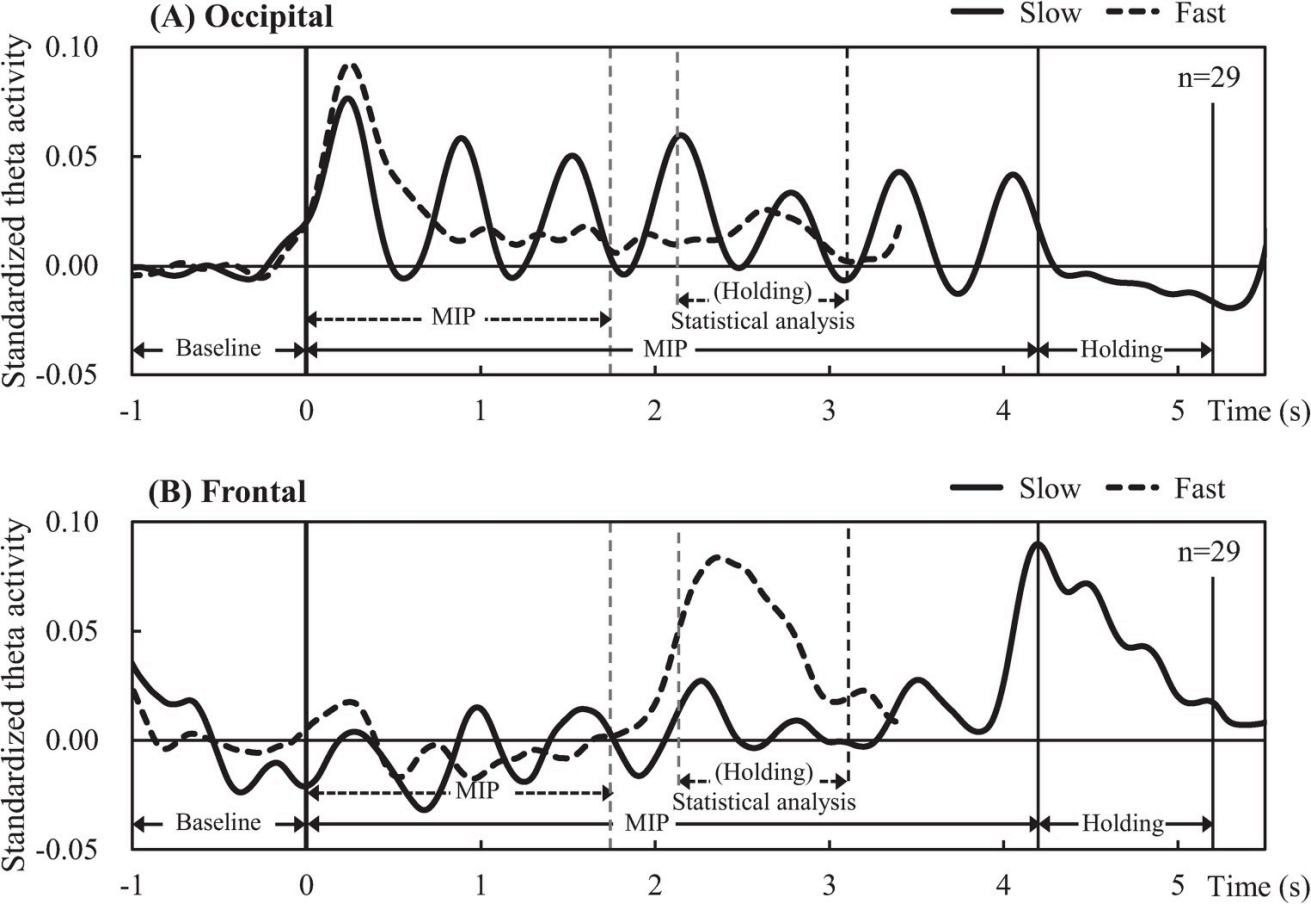
Mean time courses of standardized cortical theta activity in the target regions. Occipital-theta (A) exhibited prominent peaks after each arrow (memory item) presentation in the slow task (solid line), while exhibiting a prominent peak only after the presentation of the first arrow in the fast task (broken line). Frontal-theta (B) exhibited broad peaks in the holding period in both tasks (solid and broken lines).

In the fast task, significantly positive t-values in the occipital region were observed only during the beginning sub-period (Fig 4B). However, as described in the Methods section, target regions for further analysis were determined by considering activity across the entire MIP period and holding period and by highlighting the common vertices for the slow and fast tasks for which standardized cortical theta-rhythm activity was statistically positive above baseline. Fig 4(C) shows the resulting occipital and frontal target regions during the full MIP period and holding periods, respectively. Standardized theta activity time-courses extracted from occipital and frontal target regions are shown in Fig 5(A) and 5(B), respectively. The time-course of the slow task (solid lines in Fig 5) revealed theta synchronization in both the occipital (A) and frontal (B) regions following the presentation of each arrow. Meanwhile, the time-course of the fast task (broken lines in Fig 5) revealed marked theta synchronization in the occipital region (A) only after the presentation of the first arrow, becoming obscure following the presentation of subsequent arrows. Additionally, the theta activity time-course of the frontal region (B) in the fast task was vague during the entire MIP period. Finally, frontal-theta exhibited a strong response during the holding period in both tasks (solid and broken lines in B).

**Fig 6:**
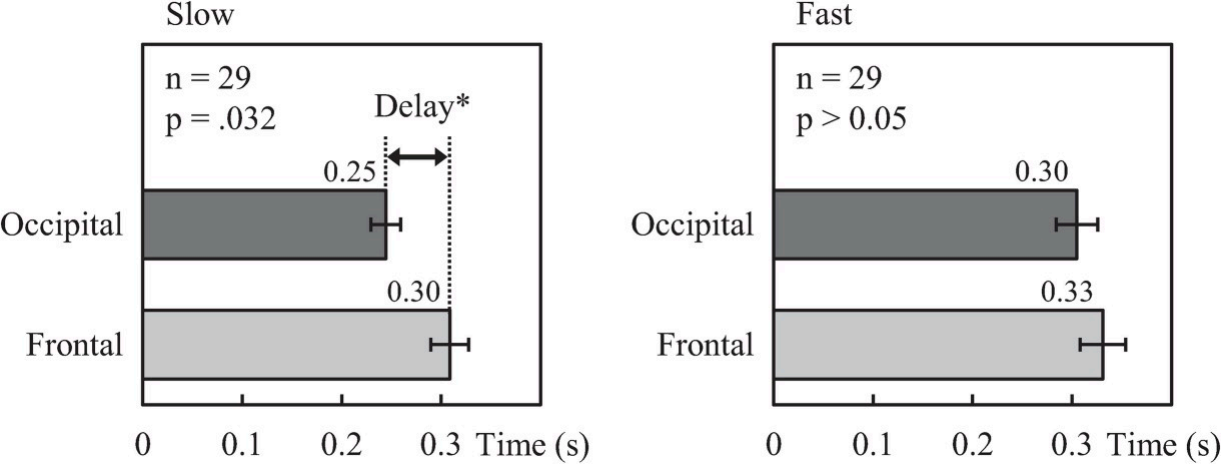
Comparison of primary peak theta amplitude timing between regions in each task. Frontal-theta amplitude peaks were significantly delayed after occipital-theta amplitude peaks in the slow task, and trending in a similar manner in the fast task, supporting the notion that frontal maintenance processing occurs downstream from visual encoding. 0 s denotes the onset of the first memory item presentation. (Error bar: SE).

As for the difference in timing between the observable theta amplitude peaks originating from the frontal and occipital regions in response to the presentation of each arrow, Table 1 summarizes the mean timing of observable peaks in each task. In the slow task, the timing of the primary frontal region peak (0.30 ± 0.02 s) was significantly later than that of the occipital region (0.25 ± 0.02 s) (*p* = 0.032). No significant differences were observed in the timing of peaks corresponding to the presentation of subsequent arrows. In the fast task, the mean timing of the primary theta amplitude peak similarly trended later in the frontal region than the occipital region, but not significantly so. Fig 6 shows the mean timing of the primary peaks in the occipital and frontal standardized theta activity time-courses in the slow and fast tasks.

**Table 1:** Timing (sec.) of peak theta activity amplitude to memory item presentation.

**Slow**	**1st** *	**2nd**	**3rd**	**4th**	**5th**	**6th**	**7th**
**Occipital**	0.25±0.02	0.31±0.02	0.29±0.02	0.34±0.02	0.32±0.03	0.36±0.03	0.40±0.03
**Frontal**	0.30±0.02	0.38±0.03	0.32±0.03	0.40±0.03	0.25±0.04	0.36±0.04	0.39±0.04
**Fast**	**1st**	**2nd**	**3rd**	**4th**	**5th**	**6th**	**7th**
**Occipital**	0.30±0.02	-	-	-	-	-	-
**Frontal**	0.33±0.03	**-**	**-**	**-**	**-**	**-**	**-**

(Mean±SE, n = 29

*significant difference between occipital and frontal: p < .05).

**Fig 7:**
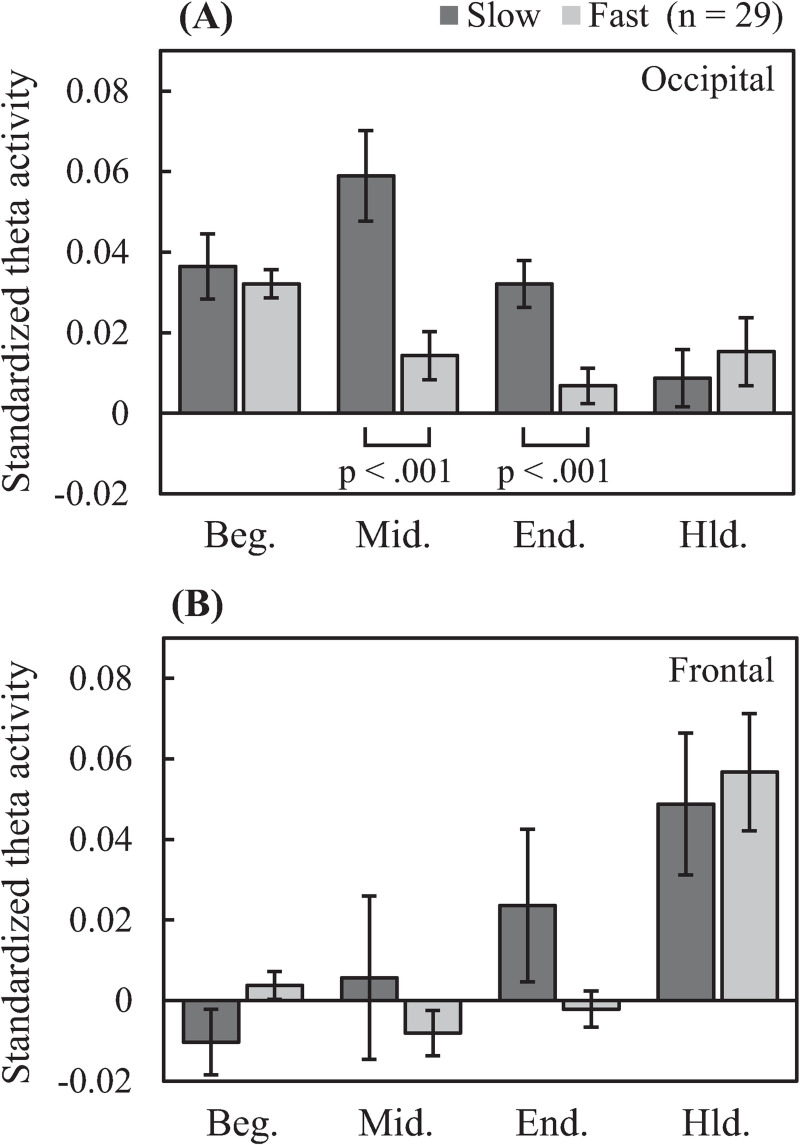
Time-integrated comparison region-specific standardized cortical theta activity between tasks according to task period/sub-period. Occipital-theta activity is greater for the slow task than the fast task in the midterm and ending sub-periods. (Beg.: beginning sub-period, Mid.: midterm sub-period, End.: ending sub-period, Hld.: holding period).

Two-way RM ANOVA of standardized cortical theta activity in the occipital region (see Fig 7A) revealed a significant interaction between task and period/sub-period (*F*_(3, 84)_ = 9.969, *p* < 0.001, *η*^2^ = 0.263). Simple main effects testing revealed that standardized cortical theta activity in the fast task was significantly lower than that in the slow task during the midterm (*p* < 0.001) and ending (*p* < 0.001) sub-periods. Meanwhile, two-way RM ANOVA of standardized cortical theta activity in the frontal region revealed a significant interaction between task and period/sub-period (*F*_(3, 84)_ = 3.883, *p* = 0.022, *η*^2^ = 0.122). However, subsequent simple main effects testing revealed no significant differences between tasks in any period/sub-period.

## 4. Discussion

This research aimed to gain new insight in the functional neural processes that underlie memory performance decline by analyzing MEG theta activity recorded during performance of a sequential memory task in which directional arrow-based memory items were presented at two different rates, slow or fast. Our analyses suggested the importance of regions in the occipital and frontal cortex for sequential memory in both the slow and fast tasks. However, the fast task resulted in declined memory performance which corresponded with declined overall theta activity levels from the midterm to the ending of the MIP period, as well as declined peak theta responsiveness to individual memory items. Meanwhile, a relationship regarding the timing of peak theta response to memory item presentation was observed between the occipital and frontal regions. Here we discuss these results in detail, beginning with memory performance.

### 4.1 Memory performance

Accuracies (Fig 2) in both tasks exhibited U-shaped curves which are typically observed in sequential memory tasks, including our previous studies [31, 32]. In other words, both the slow and the fast task exhibited serial position effects. However, the fast task resulted in a much stronger serial position effect, as evidenced by its steeper U-shaped curve, and significantly lower accuracy compared to the slow task for recall numbers corresponding to memory items three and onward. Thus, the shorter available processing time due to the shorter ISI between memory items in the fast task is clearly detrimental to memory performance, particularly during the midterm and ending sub-periods.

### 4.2 Occipital- and frontal-theta

As previous neuroimaging studies of memory performance modulation by ISI are limited, we first tried an exploratory, whole-brain approach in this work. The cortical t-maps (Fig 4), which show that standardized cortical theta activities were statistically positive above baseline, suggested the importance of regions in the occipital and frontal cortices. Although not statistically robust in terms of multiple comparisons, these results are corroborated by numerous previous works pointing to the importance of the occipital and frontal regions in working memory [4, 6–8, 13–16, 24–26, 29, 30]. Given this corroborative evidence, we considered the result of the t-maps to be valid. Hence, we extracted the target occipital and frontal regions based on the t-maps and analyzed the temporal changes in theta activities occurring therein.

#### 4.2.1 Occipital-theta

When time-integrated in each MIP sub-period (Fig 7A), standardized occipital-theta activity was significantly lower for the fast task than that of the slow task, especially in the midterm and ending sub-periods, in correspondence with significantly lower accuracy for memory items presented in the same sub-periods (Fig 2). Moreover, occipital-theta time-series waveform dynamics exhibited marked synchronization in response to the presentation of each memory item but did not appear to exhibit broad U-shaped dynamics across the MIP period as were observed with accuracy (see Fig 5A), suggesting that occipital-theta activity contributed to memory performance via a mechanism that was different from that which contributes to serial position effects. The phenomenon of increased occipital-theta levels after memory item presentation has been frequently reported [3, 37–39], and has been proposed to reflect visual processing [22, 23]. In the present study, occipital-theta in the slow task (solid line) synchronized after the presentation of every memory item, with peaks of 0.25 s or longer latencies (Table 1). Meanwhile, occipital-theta in the fast task (dashed line) synchronized strongly only after the presentation of the first memory item. Subsequently, mild synchronization is evident until the presentation onset of the third memory item, but becomes unclear thereafter. This loss of clarity in theta responsiveness to memory item presentation was in exact correspondence with the onset of significant differences in recall accuracy (from the 3rd to the final memory item). Considering that the latency of peak theta response to memory item presentation in the slow task was around 0.25 s and that the ISI of memory item presentation in the fast task was also 0.25 s, it may be that the ISI of the fast task intrinsically interfered with theta-related cognitive processing important for memory performance. Supposing that this cognitive processing is visual encoding of the memory items, the fast task resulted in clear processing deficiencies compared to the slow task. These encoding deficiencies possibly fundamentally underlie the differences in theta activity and memory performance that were observed between the slow and fast tasks. Comprehensively, this suggests that a minimum time duration between memory item presentation may be required for effective visual encoding of memory items to ensure that the information can be properly stored and maintained until recall. This minimum time duration may be around 0.5 s, which is time duration required for occipital-theta amplitude to recover to baseline after memory item presentation in the slow task of the present study (Fig 6A).

#### 4.2.2 Frontal-theta

Cortical activity maps in Fig 4(A) and 4(B) right illustrate that frontal-theta activity was predominant during the holding period in both slow and fast tasks. Indeed, frontal-theta synchronized markedly (Fig 5B) and to similar and non-significantly different extents during the holding period in both tasks (Fig 7B). Conversely, frontal-theta activities in both tasks did not increase markedly in amplitude during the MIP period, although they rose and fell modestly (Fig 5B). Some previous studies have reported that frontal-theta increases during memory maintenance [4, 7, 8, 13]. In the present study, the holding period represents the full transition to memory maintenance following the concurrent encoding and maintenance that occurs during the MIP period. In this light, our results are in line with prior reports, and demonstrate that memory maintenance processes operated well in both tasks. Additionally, frontal-theta has been shown to correlate with the number of memory items maintained [13], and to increase in correspondence to mental effort [40]. This could explain the lack of significant differences between tasks, as both tasks featured the same number of memory items, and thereby presumably required similar levels of mental effort for memory maintenance. Altogether, the frontal-theta activity results indicate that the processing burden of memory maintenance is not significantly impacted by shortening the interval of memory item presentation, thereby further implicating that performance declines due to the shortened ISI are driven by encoding processing deficiencies in occipital visual regions.

#### 4.2.3 Relationship between occipital- and frontal-theta

As mentioned above, frontal-theta activity did not markedly increase during the MIP period (Fig 5B). However, particularly in the slow task (solid line in Fig 5B), frontal-theta activity did rise and fall numerous times. In fact, the timing of this frontal-theta modulation closely followed that exhibited by occipital-theta (solid line in Fig 5A) after the presentation of every memory item. Although this memory item response phenomenon was not as clearly evident in the fast task, similar brain activity patterns in occipital and frontal regions have been previously observed during memory tasks [41], including theta activity patterns specifically [37]. As discussed in the previous paragraph, frontal-theta is thought to reflect top-down memory maintenance processing [4, 7, 8, 13]. Moreover, disruption of frontal-theta using transcranial magnetic stimulation has been shown to functionally disrupt working memory performance [42]. In our sequential memory task, new memory items are encoded while simultaneously maintaining memory items which have already been encoded (see Fig 1). Thus, frontal-theta (Fig 5B, solid line) modulation during the MIP period could be explained as reflecting an aspect of top-down memory maintenance processing related to the updating of memory, a notion supported by prior reports [7, 13]. However, the top-down response in the frontal cortex during visual memory processes is arguably driven by visual input, as was demonstrated in a monkey study by Hasegawa et al. [43].

Our results also appear to show visual input being the driving factor in frontal-theta. In the slow task, the primary peaks of theta-rhythm synchronization were significantly later in the frontal region than in the occipital region by about 60 ms (Figs 5 and 6). Delays continued in the second through fourth peaks as well (see Table 1), albeit not significantly, and then disappear by the end of the sequence. Naturally, the deterioration in the observability of delays downstream in the task can partially be attributed to increased response variability associated with lower accuracy rates, particularly for midterm memory items. However, considering that the memory items are presented rhythmically at fixed time intervals, the gradual disappearance of the observable delay in frontal after occipital-theta peak response may be more attributable to cortical entrainment, which is a well-known phenomenon of theta response to rhythmic audiovisual stimuli [44, 45]. That aside, the clear observation of a delay of frontal-theta peak response after the occipital-theta peak response, particularly at the start of the slow task, indicates that occipital processing precedes frontal processing in response to memory item presentation. This correspondingly suggests signal transmission from the occipital region to the frontal region, a notion supported by numerous reports on memory function [3, 46, 47]. Indeed, Sauseng et al. (2004) [46] proposed the importance of the interaction between the occipital region for encoding and storing sensory information, and the frontal region for updating and maintaining them. Moreover, theta is well recognized as a key frequency band for inter-cortical communication [47]. In this light, the comparative lack of consistent and clear frontal-theta response peaks to memory item presentation in the fast task could be a sign that the encoding deficiencies observed in the occipital-theta response led to a breakdown in communication between occipital and frontal regions during the MIP period. Future studies based on our sequential memory paradigm should attempt to quantitatively confirm this idea through measures of intercortical coherence or causality. Regardless, the encoding deficiencies observed in occipital-theta in the fast task coupled with the observation that occipital-theta peak responses to memory items preceded those of frontal-theta strengthens the interpretation that shortening the ISI between memory items disrupts memory performance at the level of visual encoding.

### 4.3 Limitations

There are a few limitations worth acknowledging regarding the present study. First, we attempted to clarify neural mechanisms of sequential memory performance by manipulating the rate of memory item presentation and analyzing and comparing cortical theta activity in young healthy adults. Although this led to unique insight into sequential memory processing, the generalizability of these results towards other subject demographics or to age-based sequential memory dysfunction remains to be seen. Future studies comparing aged and healthy young adults are needed to confirm the relationship between declined memory performance and altered theta activity in visual processing areas during memory encoding. Additionally, the present analyses were limited to theta-band activity. Further research regarding faster frequency bands may provide added clarity into the mechanisms observed in the present study. Finally, comparisons of theta activity between tasks considered broad time periods and did not control for the difference in memory performance between tasks. This approach was appropriate for our goal of clarifying general neural mechanisms underlying sequential memory performance. However, to gain more detailed insight into how memory presentation rate affects neural processing, future analyses would benefit from separating correct and incorrect trials and looking at event-related processing differences in response to individual memory items.

## 5. Conclusion

To neurophysiologically clarify the implicit real-world difficulty encountered when attempting to memorize a stream of information that is presented too quickly, we explored the relationship between sequential memory performance and theta-band activity recorded during fast and slow sequential working memory tasks, with shorter and longer memory item presentation intervals, respectively. Compared to the slow task, the fast task significantly eroded occipital-theta activity from the midterm to the ending of memory item presentation, in correspondence with a significant decline in recall accuracy for items in the same period, suggesting a breakdown of visual encoding. Meanwhile, a lack of significant differences in frontal-theta activity throughout both tasks, particularly during the holding period prior to recall, suggested that memory maintenance function in and of itself was not adversely affected in the fast task. Finally, an analysis of the timing of peak theta responses to the presentation of the memory items revealed a delay of frontal responses after occipital responses, corroborating prior observations that visual encoding processing involves communication with the frontal cortex to coordinate executive control of maintenance. Our results suggest that breakdowns in visual sequential memory processing due to faster memory item presentation rates begin at the stage of visual encoding, likely leading to a lack of or inaccurate information maintained in memory storage. Future research using our sequential memory paradigm should investigate the intercortical communication of visually encoded information via analyses of coherence or causality between the occipital and frontal regions.

## Supporting information

S1 Dataset Accuracy of each participant (U1–U29) according to tasks (slow/fast) and recall numbers (1–7): Supporting information of Fig 2. (PDF)

S2 Dataset Timing of the primary peak theta amplitude of the occipital and frontal regions in the slow and fast tasks: Supporting information of Table 1 and Fig 6. (PDF)

S3 Dataset Time-integrated standardized cortical theta activity of the occipital region according to tasks (slow/fast) and period/sub-periods (Beg: beginning sub-period, Mid: midterm sub-period, End: ending sub-period, Hld: holding period): Supporting information of Fig 7(A). (PDF)

S4 Dataset Time-integrated standardized cortical theta activity of the frontal region according to tasks (slow/fast) and period/sub-periods (Beg: beginning sub-period, Mid: midterm sub-period, End: ending sub-period, Hld: holding period): Supporting information of Fig 7(B). (PDF)
