# Supplementary material for: Processing time affects sequential memory performance beginning at the level of visual encoding
Source: PLoS One. 2022 Mar 23;17(3):e0265719. doi: 10.1371/journal.pone.0265719 (PMC8942227; doi:10.1371/journal.pone.0265719)
Supplement: S1 Dataset — (PDF) [file pone.0265719.s001.pdf]

	Slow							Fast						
Sub	1	2	3	4	5	6	7	1	2	3	4	5	6	7
U01	0.875	0.6875	0.8125	0.5625	0.5625	0.6875	0.8125	0.75	0.8125	0.75	0.8125	0.5625	0.5625	0.875
U02	0.3125	0.5625	0.25	0.4375	0.5	0.8125	0.875	0.625	0.125	0.3125	0.5625	0.625	0.75	1
U03	0.9375	0.8125	0.875	0.6875	0.875	0.6875	0.9375	0.6875	0.625	0.5	0.4375	0.5625	0.9375	0.9375
U04	0.8125	0.875	0.875	0.75	0.75	0.5625	0.625	0.875	0.8125	0.875	0.6875	0.5	0.5	0.75
U05	0.8125	0.75	0.75	0.6875	0.875	0.8125	0.8125	0.5625	0.625	0.25	0.6875	0.625	0.9375	0.875
U06	0.875	0.9375	0.9375	0.625	0.625	0.5	0.875	0.8125	0.9375	0.875	0.625	0.4375	0.4375	0.8125
U07	0.6875	0.75	0.75	0.625	0.5625	0.8125	0.875	0.875	0.875	0.6875	0.4375	0.5	0.6875	0.625
U08	0.75	0.75	0.5625	0.75	0.625	0.8125	0.8125	0.8125	0.6875	0.6875	0.6875	0.625	0.5	0.5
U09	0.9375	0.6875	0.5625	0.9375	0.9375	0.75	1	0.8125	0.8125	0.375	0.625	0.9375	0.75	0.875
U10	0.625	0.5	0.5	0.5	0.4375	0.5625	0.9375	0.6875	0.5625	0.3125	0.25	0.25	0.6875	0.75
U11	0.8125	0.875	0.8125	0.8125	0.8125	0.75	0.8125	0.9375	0.9375	0.75	0.6875	0.375	0.375	0.8125
U12	0.9375	0.875	0.8125	0.625	0.5625	0.6875	0.75	0.875	0.875	0.6875	0.75	0.5625	0.375	0.625
U13	1	0.75	1	0.875	0.6875	0.875	0.9375	0.75	0.625	0.75	0.6875	0.4375	0.6875	0.9375
U14	0.6875	0.75	0.75	0.4375	0.75	0.8125	1	0.625	0.75	0.5	0.4375	0.625	0.8125	0.875
U15	0.9375	0.8125	0.9375	0.8125	0.75	0.6875	0.6875	0.8125	1	0.5625	0.5	0.3125	0.3125	0.8125
U16	0.625	0.75	0.8125	0.5625	0.8125	0.9375	1	1	0.75	0.5	0.6875	0.5625	0.875	0.8125
U17	1	0.8125	1	0.875	0.875	1	0.875	0.75	0.8125	0.8125	0.8125	0.75	0.8125	0.9375
U18	0.9375	0.8125	0.875	0.75	0.5	0.5	0.6875	0.9375	0.9375	0.875	1	0.25	0.375	0.375
U19	0.8125	0.9375	0.5625	0.6875	0.6875	0.875	0.9375	0.9375	0.5	0.5625	0.375	0.5	0.625	1
U20	0.8125	0.75	0.5	0.5	0.4375	0.9375	1	0.8125	0.8125	0.5	0.4375	0.4375	0.625	0.75
U21	0.75	0.6875	0.8125	0.4375	0.375	0.6875	0.875	0.75	0.375	0.25	0.25	0.5625	0.5	0.9375
U22	0.875	0.8125	0.75	0.5625	0.5	0.8125	1	0.6875	0.9375	0.625	0.6875	0.8125	0.75	1
U23	1	0.8125	0.875	0.8125	0.75	0.8125	0.8125	1	0.875	0.9375	0.625	0.625	0.375	0.6875
U24	1	0.9375	0.8125	0.8125	1	0.875	0.8125	0.9375	0.875	0.75	0.6875	0.4375	0.875	0.9375
U25	0.9375	0.8125	0.8125	0.875	0.375	0.75	0.75	0.875	0.6875	0.5625	0.6875	0.5	0.4375	0.75
U26	0.5	0.5	0.5625	0.4375	0.3125	0.6875	0.9375	0.8125	0.5	0.375	0.25	0.375	0.5625	0.6875
U27	0.625	0.5	0.375	0.1875	0.5	0.6875	0.75	0.625	0.25	0.25	0.4375	0.3125	0.625	0.625
U28	0.6875	0.5625	0.6875	0.875	0.5	0.75	0.9375	0.6875	0.5	0.5	0.5	0.625	0.625	0.8125
U29	0.8125	0.75	0.5	0.4375	0.625	0.5625	0.6875	1	0.875	0.6875	0.5625	0.4375	0.5625	0.6875
