# Supplementary material for: Processing time affects sequential memory performance beginning at the level of visual encoding
Source: PLoS One. 2022 Mar 23;17(3):e0265719. doi: 10.1371/journal.pone.0265719 (PMC8942227; doi:10.1371/journal.pone.0265719)
Supplement: S2 Dataset — (PDF) [file pone.0265719.s002.pdf]

SLOW

Sub	Occipital (s)	Frontal (s)
U01	0.25	0.23
U02	0.28	0.3
U03	0.36	0.44
U04	0.03	0.33
U05	0.2	0.33
U06	0.17	0.35
U07	0.34	0.44
U08	0.3	0.22
U09	0.29	0.38
U10	0.22	0.13
U11	0.25	0.23
U12	0.13	0.17
U13	0.27	0.3
U14	0.11	0.45
U15	0.23	0.41
U16	0.29	0.29
U17	0.25	0.44
U18	0.23	0.33
U19	0.21	0.32
U20	0.24	0.12
U21	0.22	0.29
U22	0.15	0.21
U23	0.23	0.25
U24	0.36	0.18
U25	0.33	0.23
U26	0.24	0.46
U27	0.21	0.43
U28	0.39	0.19
U29	0.37	0.33

FAST

Sub	Occipital (s)	Frontal (s)
U01	0.3	0.26
U02	0.35	0.35
U03	0.46	0.52
U04	0.04	0.35
U05	0.24	0.37
U06	0.15	0.35
U07	0.42	0.5
U08	0.37	0.34
U09	0.27	0.39
U10	0.27	0.18
U11	0.31	0.32
U12	0.19	0.21
U13	0.36	0.35
U14	0.1	0.41
U15	0.3	0.39
U16	0.34	0.3
U17	0.21	0.4
U18	0.22	0.39
U19	0.27	0.33
U20	0.31	0.22
U21	0.32	0.33
U22	0.23	0.27
U23	0.22	0.29
U24	0.38	0.22
U25	0.4	0.24
U26	0.37	0.39
U27	0.31	0.38
U28	0.49	0.24
U29	0.43	0.37
