# Supplementary material for: Processing time affects sequential memory performance beginning at the level of visual encoding
Source: PLoS One. 2022 Mar 23;17(3):e0265719. doi: 10.1371/journal.pone.0265719 (PMC8942227; doi:10.1371/journal.pone.0265719)
Supplement: S3 Dataset — (PDF) [file pone.0265719.s003.pdf]

	Slow (× 10^{-2})				Fast (× 10^{-2})			
Sub	Beg	Mid	End	Hld	Beg	Mid	End	Hld
U01	-3.53389	-2.84246	1.04635	-0.97681	-0.08417	-5.27565	-1.78085	3.253349
U02	-7.33589	-1.86599	0.351281	-3.49881	0.224642	-0.39405	0.907867	2.931483
U03	-8.92179	-8.94294	-5.81605	-6.23584	-1.47356	-4.7774	-0.81297	-7.59017
U04	-5.40238	-10.7074	-4.51592	-2.37357	-2.05659	-6.26663	-4.093	-6.13912
U05	-0.61982	-1.42669	1.586938	3.980685	3.41357	1.684065	-1.4007	6.175979
U06	6.866424	6.993265	7.212319	6.54594	-0.04608	-1.27182	0.074275	5.387694
U07	1.653524	6.771468	10.26198	26.31159	2.795595	2.526303	3.879245	14.46096
U08	-3.95027	-16.9319	-9.58168	-6.35426	-1.01307	-0.91475	-0.5788	4.817964
U09	-1.68761	1.082683	-0.59413	10.5198	1.623003	2.278646	1.306271	6.608753
U10	-0.22098	5.233017	3.551134	6.773365	1.083365	1.985294	0.903776	7.298303
U11	3.734106	9.364812	8.722501	3.791098	3.329095	-6.2688	-4.32372	-5.64644
U12	-2.10241	-3.12156	-0.24337	-0.06533	0.758152	0.002462	0.224701	2.48427
U13	-6.11215	0.421965	0.178382	1.18493	0.424202	0.32546	1.47845	9.217778
U14	11.10701	18.55588	16.50572	10.93416	-0.56512	-0.00068	0.392945	11.73822
U15	0.123452	2.160468	8.063097	2.465554	1.741892	3.00009	1.525119	4.220029
U16	-2.75938	-6.71707	-5.79191	-0.56398	-1.35879	-1.80667	0.359405	6.536165
U17	2.759691	6.513582	13.26181	11.7223	3.809843	-0.44811	1.552303	8.512749
U18	-2.50736	-7.21868	-5.57878	-4.16011	0.162598	0.134863	-1.17558	-1.20263
U19	-1.68579	-2.91804	-5.45007	-1.05634	2.036018	0.889743	-1.26878	-1.11803
U20	5.316714	12.69421	6.19432	9.926576	1.569089	3.213967	-1.36953	11.92415
U21	3.185032	-0.07144	6.239693	14.69261	-2.50828	-6.15053	-3.8534	23.54645
U22	-1.20654	-3.54416	-5.21823	-1.72822	-0.09517	-3.3653	-2.44282	-0.22186
U23	10.36126	14.71784	14.81066	8.779252	-0.94198	1.912303	5.086038	6.807678
U24	-9.48441	-17.6075	-10.3918	0.414115	-1.56315	-2.44344	-3.0192	10.35868
U25	2.27764	29.56153	35.46847	30.52327	1.485388	3.025469	2.364182	23.19994
U26	-1.41356	2.011554	4.177446	22.19428	-0.55996	1.174636	2.289488	16.85965
U27	-14.3194	-23.3043	-21.3881	-10.6025	-1.24339	-4.7399	-4.76772	-8.82817
U28	-0.51185	11.17234	2.562104	7.828328	-1.4376	-3.90182	-0.64144	1.201483
U29	-3.46805	-3.57027	2.728997	0.480507	1.409641	2.501151	2.972934	7.691217
