# Supplementary material for: Processing time affects sequential memory performance beginning at the level of visual encoding
Source: PLoS One. 2022 Mar 23;17(3):e0265719. doi: 10.1371/journal.pone.0265719 (PMC8942227; doi:10.1371/journal.pone.0265719)
Supplement: S4 Dataset — (PDF) [file pone.0265719.s004.pdf]

	Slow (×10^{-2})				Fast (×10^{-2})			
Sub	Beg	Mid	End	Hld	Beg	Mid	End	Hld
U01	-3.99221	-5.23824	2.913103	-8.31538	-0.6022	-4.87227	-3.46277	-1.97405
U02	4.082824	4.99097	2.012566	-1.56539	3.959757	1.298317	0.143	-2.02865
U03	1.449132	0.536583	-0.09115	0.998859	0.27606	0.371758	0.242414	-1.40234
U04	-2.02916	6.555108	5.708038	-6.73426	2.023082	-6.80768	-4.89157	-6.24036
U05	1.255164	-2.36484	3.256757	2.761575	7.679198	4.633901	1.589876	8.735029
U06	5.55738	3.444034	3.062842	-0.26089	2.435377	-0.2877	-1.2776	-2.34673
U07	-1.96656	-2.03634	-1.01609	-0.69647	2.931856	1.734639	-0.12793	-1.60301
U08	-4.24929	9.806614	5.010605	11.19441	3.204884	1.835747	0.956087	8.999636
U09	2.874945	0.483802	-2.83658	-1.42629	0.778725	-0.63681	-2.05167	-3.30304
U10	7.620624	18.51219	9.771333	2.138465	5.955186	10.38954	6.219211	3.932864
U11	11.28223	21.1128	10.67998	3.877281	4.721026	0.061878	2.259198	16.52204
U12	6.103036	2.08662	1.86842	3.071996	2.101958	-0.88018	-1.53901	-2.36016
U13	-3.51654	3.35259	2.739197	-3.93213	1.358814	-1.03572	0.131348	0.797139
U14	5.483564	6.479224	4.936388	3.116444	1.955824	3.32895	2.882838	4.014077
U15	7.919802	10.19924	4.129451	0.709179	5.552882	4.545074	1.753797	1.528055
U16	8.807388	16.17945	3.867955	1.195538	5.388266	6.506892	3.577278	1.298226
U17	7.548527	9.229353	4.173221	0.778362	3.060696	2.771174	-0.07199	0.094869
U18	4.701197	2.006377	5.619204	1.979199	2.581731	1.443103	2.093349	3.794464
U19	7.616986	4.585833	2.952966	1.726484	4.536161	1.750656	-0.01103	-2.09113
U20	5.381483	5.837312	2.633634	2.249179	4.248717	1.456328	-1.09045	2.553644
U21	11.78628	16.94337	8.195005	6.152781	3.806971	4.509585	6.227142	7.79252
U22	5.211355	4.005082	-1.44836	-3.97273	2.180456	-0.41516	1.085188	-2.01525
U23	6.279589	7.675269	4.22852	-1.02504	5.107404	2.574324	1.526393	-0.55452
U24	-2.40153	5.3425	-0.71265	-0.16028	2.245609	0.683498	0.872829	2.521161
U25	2.031839	5.411202	5.019304	0.645285	2.06812	0.374952	-0.32756	2.46844
U26	4.506741	5.392252	4.344267	7.313523	4.979892	2.462342	1.335584	4.596745
U27	-0.72207	1.416981	-1.19677	-1.22801	1.132405	-2.21638	-1.65216	-0.88622
U28	4.617788	7.463321	-0.15913	0.957444	1.903806	1.48041	1.067106	0.630431
U29	2.450698	1.57404	3.507683	3.844423	5.556048	4.471861	2.385125	0.946393
